# Evaluation of a toolbox for the prevention of skin cancer among outdoor workers: an intervention study

**DOI:** 10.3389/fpubh.2025.1579180

**Published:** 2025-06-09

**Authors:** Florentine L. de Boer, Daisy Vreenegoor, Jos W. R. Twisk, Jack J. van der Gragt, Thomas Rustemeyer, Sanja Kezic, Henk F. van der Molen

**Affiliations:** ^1^Department of Public and Occupational Health, Amsterdam Public Health Research Institute, Amsterdam UMC, University of Amsterdam, Amsterdam, Netherlands; ^2^Department of Epidemiology and Data Science, Amsterdam University Medical Centers, Amsterdam, Netherlands; ^3^Volandis, Harderwijk, Netherlands; ^4^Department of Dermatology and Allergology, Amsterdam University Medical Centers, Amsterdam, Netherlands

**Keywords:** non-melanoma skin cancer, solar radiation, ultraviolet exposure, construction workers, arboricultural workers, occupational disease, sunscreen use, stratum corneum

## Abstract

**Introduction:**

Due to ultraviolet radiation (UVR) exposure at work, outdoor workers face a higher risk of keratinocyte carcinoma (KC) than indoor workers. This study evaluates the short-term effectiveness of a sun-safety risk communication toolbox aimed to increase sun-safety behavior among male outdoor workers.

**Methods:**

This parallel-controlled, non-randomized study included outdoor construction and arboricultural workers, recruited from five companies. Twenty-eight workers were assigned to the intervention group, where they received a preventive toolbox, while 26 workers were assigned to the control group. The toolbox included information on UVR health risks and preventive measures, as well as sunscreen provision. The primary outcome was internal UVR exposure, measured by the relative cis-urocanic acid (cUCA) levels in the stratum corneum (SC). SC samples were taken from two skin sites (cheek and neck) at baseline and 6 weeks. Secondary outcomes included sun-protective behavior, workplace encouragement, knowledge and attitude/motivation, all assessed using questionnaires.

**Results:**

A difference in cUCA was found between groups with lower cUCA at the cheek (−0.065 (95% CI: −0.101 to −0.029)) and neck location (−0.032 (95% CI: −0.068–0.004)) for the intervention group. Reported sunscreen use significantly improved in the intervention compared with control group (difference between group (11.01 (95% CI: 2.04–20.10)). For other secondary outcomes no statistical differences between groups were found.

**Conclusion:**

The toolbox intervention led to a reduction of internal UVR exposure, consistent with a self-reported increase in sunscreen use, compared to no intervention. Future research should focus on the longer-term preventive effects of this type of toolbox following further development and evaluation.

## 1 Introduction

Keratinocyte carcinoma (KC), formerly known as non-melanoma skin cancer, is the most common malignancy worldwide with rapidly rising incidence (1, 2). KC includes basal cell carcinoma (BCC) and cutaneous squamous cell carcinoma (cSCC), both contributing to significant morbidity and healthcare costs (3, 4). Solar ultraviolet radiation (UVR) is the leading cause of KC, responsible for 90% of total KC cases (5). In Europe, UVR is recognized as the most common occupational carcinogen, affecting 36 industries and leading in 11 economic sectors such as construction workers, gardeners and livestock breeders (6). Outdoor workers face a threefold higher risk of developing KC than indoor workers due to higher UVR exposure (5). The World Health Organization and the International Labour Organization have acknowledged the causal relationship between KC and occupational UVR exposure. In several European countries, KC is recognized as an occupational disease (6–8). These findings underscore the need for preventive strategies for outdoor workers exposed to high occupational UVR levels (9).

While reducing UVR exposure can lower KC risk, sun-safety behaviors are often inadequate due to organizational and personal barriers as limitations to work in the shade, no offer of protective work clothing, or indifference or resistance to apply sunscreen, (10–13). These barriers highlight the need for targeted and feasible interventions (13), reduce the risk of sunburns and skin cancer (14, 15).

Outdoor workers—who are often in lower socioeconomic positions—may have lower health literacy and varying levels of education, underlying the need for practical messaging, and use of visual aids to increase understanding of the UVR related health risks and the necessity to protect themselves (16, 17). A systematic review by Reinau et al. (10) found that occupational sun safety programs can enhance sun-protective behavior among outdoor workers. However, the authors also emphasized the need for additional investigations across different outdoor occupational groups and geographical regions, with longer follow-up periods, to identify the most effective methods of intervention and assess their impact on health outcomes related to UVR exposure. A review by Horsham et al. (18) reported that various sun safety interventions on outdoor workers show limited or no improvements in sun protection among outdoor workers.

In the Dutch construction and green sectors, ‘toolbox meetings’ are required by law and regularly held to educate workers on safety and health, addressing various workplace risks and preventive measures (19). Therefore, these toolbox meetings are good opportunities to share sun safety messaging and education and even facilitation of personal protection by provision of sunscreen.

This study aims to evaluate the short-term effectiveness of a sun safety toolbox for construction and arboricultural workers in the Netherlands. The toolbox includes an educational film on health risks of UVR, covering both preventive measures and curative options as the removal of (pre)malignant lesions, followed by an interactive discussion. Additionally, workers are provided with sunscreen, an individual-level protective strategy that is not hindered by organizational constraints.

## 2 Methods

### 2.1 Design and setting

**Figure 1:**
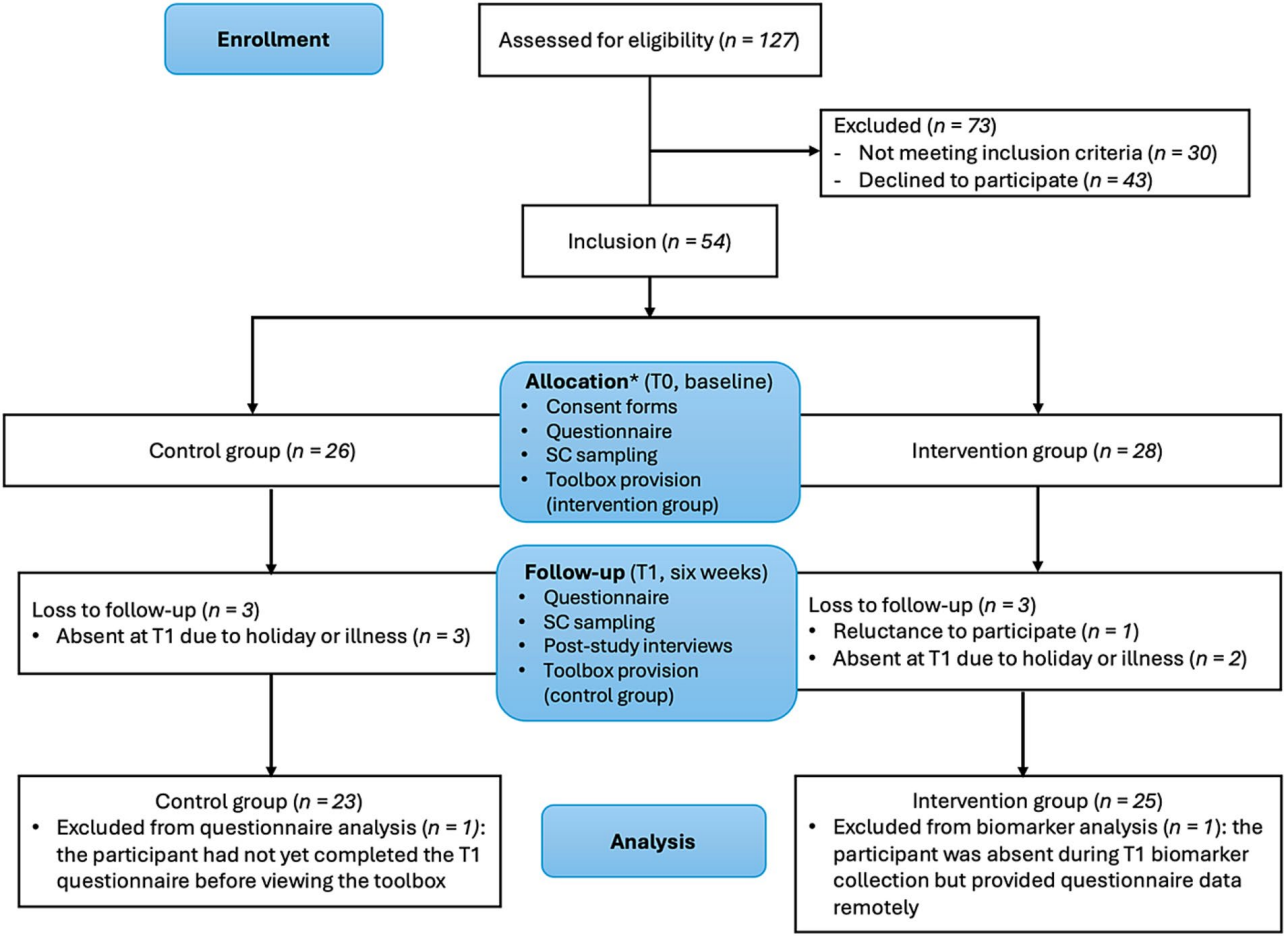
Flowchart of participants and study design. *Non-random allocation with sectors equally distributed between intervention and control group with no prior knowledge about individual participant characteristics. T0: baseline, T1: six weeks after baseline, SC: stratum corneum.

This parallel-controlled, non-randomized study was conducted from May to July 2024 in North-Holland province from The Netherlands. Outdoor workers from construction and arboricultural companies were eligible to participate. Workers were assigned to either the intervention or control group, with both groups completing a questionnaire and undergoing stratum corneum (SC) sampling at baseline (T0) and after 6 weeks (T1). The intervention group received the toolbox intervention after T0, while the control group received it after T1 to provide equal overall benefits for all participants. A process evaluation, following the Linnan & Steckler model assessed recruitment, reach, dose delivered/received, fidelity, intervention satisfaction, and context (Supplementary material S2) (1). Additionally, four participants from the intervention groups and their managers were interviewed after completion of the study. The study adhered to the Declaration of Helsinki (2013) and was approved by the Medical Ethics Committee of the Academic Medical Centre, Amsterdam. See Figure 1 for the study design and flowchart.

### 2.2 Participants and recruitment

Male construction and arboricultural workers aged 18–66 with Fitzpatrick skin phototypes II-V (2), who worked outdoors for at least 4 h daily, were included. Exclusion criteria were non-Dutch speakers, excessive UVR exposure within 6 weeks prior to the study, and presence of the lesions or skin diseases at sampling locations. Female participants were excluded from participating in this study, as construction workers were predominantly male, and the inclusion of females could potentially lead to bias in one of the groups.

#### 2.2.1 Power calculation

The sample size calculations were based on the sample size of a study with a comparable study design where the exposure to UVR was measured by self-reported UVR exposure and the difference between baseline and after the intervention was evaluated in a control (‘basic’) and intervention (‘enhanced’) group (20). We expect the intervention group to have significant decrease in UVR exposure in comparison to the control group after the intervention. In the example study from Hiemstra et al. the difference in means between baseline and the end of the intervention was 0.13 for the control group, and 0.25 for the intervention group. Based on a significance level of 0.05 and 80% power, a minimum of 15 participants per group was required (20). Taking into account potential drop-outs as seen previously within this demographic group (4), and the inclusion of two skin locations, the target was to include 30 participants per group.

#### 2.2.2 Inclusion

Eventually, 54 participants were included: 28 in the intervention group and 26 in the control group. Workers from the same company were assigned to the same group to avoid contamination bias, with the different sectors (arboricultural work, ground-and demolition work and carpentry) evenly distributed between both groups. Participating companies were recruited by the researchers through direct contact with individual contractors via telephone calls or email correspondence. The companies were selected using a convenience sampling approach, partly based on their participation in previous research and partly due to their location within the same regional area. Five companies participated: the intervention group included one construction company and one that combined construction and arboricultural work, while the control group originated from two construction and one arboricultural company. Study details were shared with the management and workers through emails, flyers, and meetings. Eligible participants enrolled in the study after providing written informed consent. Full recruitment details are given in Figure 1.

### 2.3 Study procedures

#### 2.3.1 Questionnaire

Self-reported data was collected at baseline (T0) and after 6 weeks (T1) using a Dutch questionnaire developed in prior research, with minor adjustments to fit this study design (4, 5). Three control group participants received verbal assistance due to mild language barriers or literacy issues. The questionnaire covered sociodemographic details, occupational features, and four subcategories regarding sun safety: sun-protective behavior, workplace encouragement, knowledge and attitude/motivation. The questionnaire consisted of different question formats, including binary (*yes/no*), ternary (*yes/no/I do not know* and *yes/no/sometimes*), multiple select, and single select multiple choice questions. Additionally, the T1 questionnaire included a post intervention evaluation on sunburn frequency and toolbox satisfaction. Full questionnaires are available upon request.

#### 2.3.2 Skin phototype determination

Skin phototype was determined by the researchers using the Fitzpatrick system (I-VI). The Fitzpatrick system classifies skin type based on pigmentation and its tendency to burn or tan after sun exposure (2). The assessment was based on observation of the constitutive skin and standard questions on tendency of the skin to burn and was performed by same researcher (DV) for all participants (21, 22).

#### 2.3.3 Toolbox

The toolbox included an educational film about both prevention strategies as well as the health risks of UVR exposure, followed by an interactive discussion. In addition, the participants received sunscreens. Based on outcomes of a previous study (4), construction workers helped design the film. The brief, yet comprehensive film (1 min and 37 s) portrays a typical workday of a middle-aged Caucasian construction worker. It shows colleagues encouraging him to seek medical attention for a suspicious skin spot, which leads to treatment for a pre-malignant lesion at an academic medical hospital. The film also features on-screen dermatological advice on sun protection displayed, by highlighting the importance of shade seeking, wearing sun protective clothing and the correct use of sunscreen. At baseline, the intervention group watched the film, followed by an interactive discussion led by the researcher. During the discussion, proper sunscreen application was explained, including the recommended frequency of (re)application and commonly overlooked skin areas such as the neck and retroauricular region. Participants received PGP® sunscreens (23), including two 100 mL UV 30 SUN tubes, two 100 mL UV 50 PLUS tubes, one 200 mL UV 50 PLUS spray, and one 20 mL UV 50 PLUS tester tube, ensuring adequate application for 6 weeks, with instructions to contact researchers if supplies ran low.

#### 2.3.4 Sample collection

**Figure 2:**
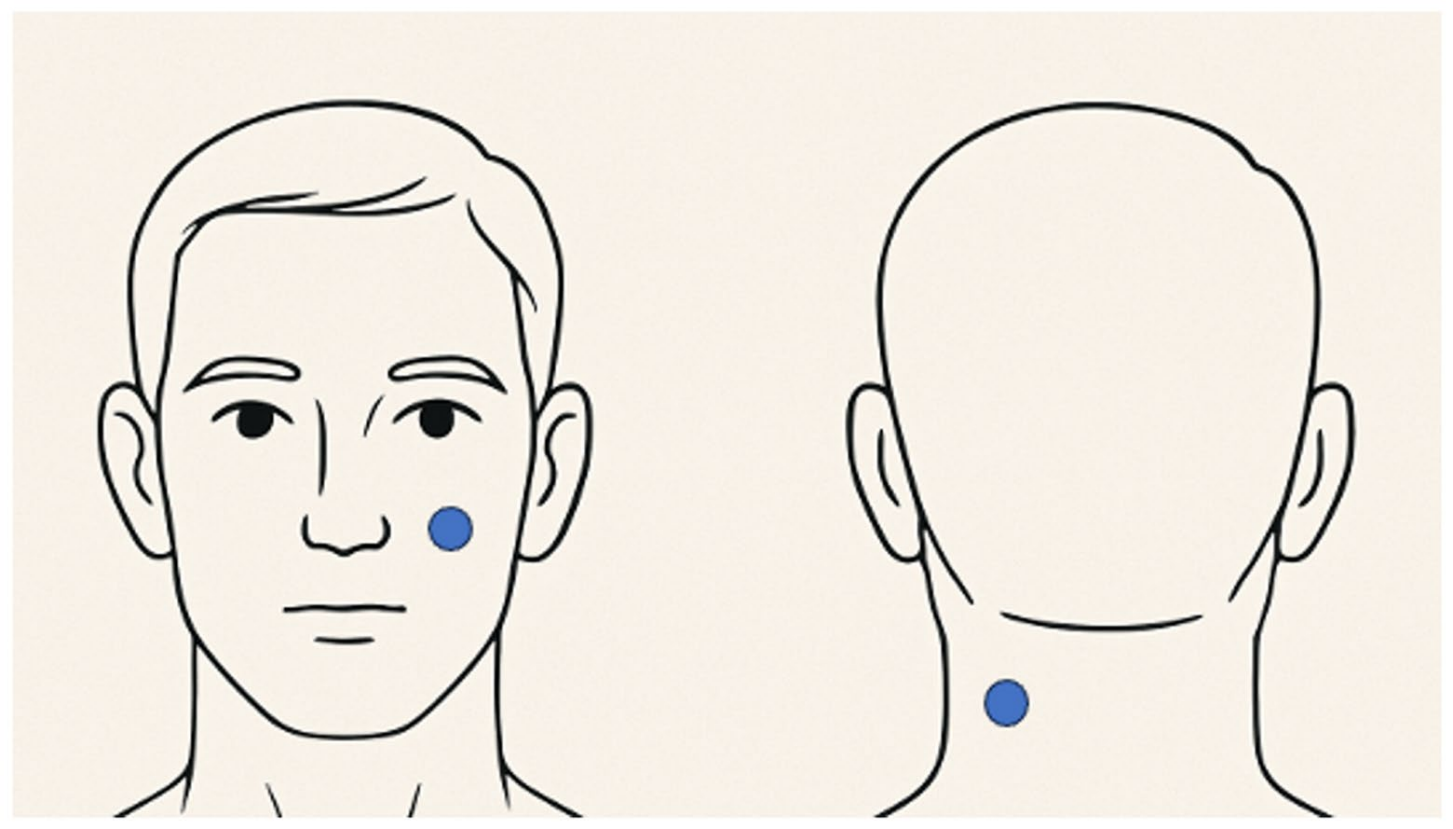
Sampling locations (7). Male head illustration generated by DALL E3 (open AI).

Non-invasive tape stripping for SC sampling was conducted using adhesive tape discs (22 mm diameter, D-Squame, CuDerm). Three sequential strips were taken from the same skin spot on cheek and neck (Figure 2). Each tape was pressed against the skin for 5 s using a Monaderm standardized pressure device (150 g/cm^2^), then removed with tweezers and placed in individual vials. The first strip was discarded, while the second and third were retained for cis urocanic acid (cUCA) analysis (24). Samples were stored at −80°C until analysis.

### 2.4 Urocanic acid analysis

Urocanic acid (UCA) isomers were quantified as described elsewhere (24). Briefly, cis-and trans-UCA isomers (cUCA and tUCA, respectively) were extracted from the tapes using 600 μL of Millipore water and analyzed by high-performance liquid chromatography with a UVR detector. The relative cUCA levels were calculated by dividing cUCA by total UCA (cUCA + tUCA).

### 2.5 Outcome measures

#### 2.5.1 Primary outcome

The effectiveness of the toolbox intervention was assessed by measuring internal UVR exposure, determined from the relative amount of cUCA collected from the cheek and neck. These body locations are particularly vulnerable to KC due to high sun exposure (25), however the neck is often overlooked during sunscreen application (26–28). Relative cUCA has been identified in previous studies as a reliable biomarker for UVR exposure (15, 28, 29). The primary outcome was the relative cUCA level at two skin locations, i.e., cheek and neck.

#### 2.5.2 Secondary outcomes

Secondary outcomes included self-reported data obtained from two questionnaires (T0 and T1). Baseline data included sociodemographic information of age, duration of tenure in their current profession, primary outdoor job tasks, and working hours spent outdoors. Four behavioral outcomes were assessed: sun-protective behavior, workplace encouragement, knowledge and attitude/motivation. Additionally, the T1 questionnaire included a post intervention evaluation regarding the frequency of sunburns during the study period and satisfaction with the toolbox. The sum scores of the categories workplace encouragement, knowledge and attitude/motivation were clustered and compared between intervention and control group (Table 1).

**Table 1:** Intervention versus control sum scores and differences between groups over time.

Questionnare category	Group	(*n*=)	T0	T1	Effect (95% CI)	*p*-value
Sun-protective behavior (0–100)	Intervention	(25)	35	50	11.01 (2.04–20.10)	0.02
	Control	(23)	27	33		
Workplace encouragement (0–100)	Intervention	(25)	44	45	5.69 (−18.13–29.50)	0.59
	Control	(23)	33	36		
Knowledge (0–100)	Intervention	(25)	33	34	4.09 (−19.13–27.31)	0.72
	Control	(23)	23	33		
Attitude/Motivation (0–100)	Intervention	(25)	55	67	5.94 (−25.60–37.49)	0.70
	Control	(23)	47	52		

This table shows the effect of the intervention with 95% confidence interval and *p*-value for the secondary outcomes: questionnaire categories Sun-protective behavior, Knowledge, Attitude/Motivation and Workplace encouragement.

##### 2.5.2.1 Sun-protective behavior

Sun-protective behavior (Cronbach’s alpha: 0.67) was evaluated through six questions about (i) the frequency and extent of the use of sunscreen, (ii) the use of sun-protective clothing such as helmets and clothing, and (iii) seeking shade. All questions from this category are described in Supplementary Table S1. The questionnaire category ‘Sun-Protective Behavior’ was scored, with each question yielding up to two points, with higher scores indicating more frequent or extensive sun-protective behavior. A sum score (0–12) was then calculated from these six questions. A supplementary scoring key is provided in Supplementary material S1.

##### 2.5.2.2 Workplace encouragement and facilitation of sun protection

Workplace encouragement (Cronbach’s alpha: 0.66) consisted of three questions about the facilitation of (i) the use of sunscreen (ii) the use of sun-protective clothing, and (iii) seeking shade (e.g., ‘*the use of sunscreen is encouraged in my workplace*’). To calculate sum scores, the questions were categorized. The answering categories were clustered to Yes/No (2 points per correct answer). A sum score (0–6) of the answers across these three questions was calculated. All questions from this category are described in Supplementary Table S1.

##### 2.5.2.3 Knowledge of sun-protective behavior and risk factors

Knowledge (Cronbach’s alpha at baseline: 0.49) was assessed through five questions related to (i) correct sunscreen application and (ii) skin cancer risk awareness (e.g., ‘*application of sunscreen is also necessary if your skin is already tanned*’ or ‘*sun exposure is the primary cause of skin cancer*’). To calculate sum scores, responses were categorized, with answer categories clustered as correct (2 points) or incorrect/do not know (0 points). A sum score (0–10) was then calculated from these five questions. All questions in this category are described in Supplementary Table S1.

##### 2.5.2.4 Attitude/motivation towards sun protection

Attitude/Motivation (Cronbach’s alpha at baseline: 0.74) consisted of six questions related to (i) attitudes towards sunscreen use and sun-protective clothing, and (ii) motivators for exhibiting sun-protective behavior (e.g., ‘*it motivates me to use sunscreen when my colleagues use it as well*’). To calculate sum scores, responses were categorized. Four questions had Yes/No options, with 2 points for correct answer. For the remaining two questions the responses were clustered into two categories: category one (2 points) for the answering options ‘Decided to use it’ and ‘Already use it’, while category two included ‘Never thought about it’, ‘Unsure if I will use it’, and ‘Decided not to use it’. A sum score (0–12) was then calculated from the right answers across all six questions. All questions from this category are described in Supplementary Table S1.

### 2.6 Statistical methods and analysis

Data were analyzed using IBM SPSS Statistics for Windows (version 28.0) and RStudio software (version 2024.04.2 + 764). To assess the normality of the residuals, histograms of the residuals were used. Descriptive statistics were reported for all variables and two-sided *p*-values <0.05 were considered statistically significant.

#### 2.6.1 Primary outcome: biomarkers

To estimate the difference in relative cUCA between the intervention and control group we applied a linear mixed model (LMM) analysis to account for the correlated observations across the two skin locations (cheek and neck). LMM analysis was also applied to investigate the differences within the groups. To estimate the difference between the two groups at both locations, an interaction between group and location was included in the model. Additionally, an adjustment was made for the baseline value of the outcome variable.

#### 2.6.2 Secondary outcomes: sun protective behavior, workplace encouragement, knowledge, attitude/motivation assessed by questionnaires

Only participants with complete data at baseline and at the end of the intervention (T0 and T1, respectively) were included in the statistical analysis. The answers from the questionnaire categories were dichotomized or placed on a scale, and the sum scores were converted into continuous variables on a scale of 0–100 (sun-protective behavior, knowledge, attitude/motivation, workplace encouragement). To analyze the differences between the intervention and control group over time, LMM analysis for the sum scores was performed.

## 3 Results

At baseline, 54 participants were included in the study. Loss to follow-up was 11% in the intervention group, and 12% in the control group, leaving 25 participants in the intervention group and 23 in the control group (Figure 1). After baseline data collection, one participant in the intervention group was excluded from participation due to reluctance to participate. The remaining five participants were lost to follow-up, as they were absent at the second appointment due to illness or holidays. Additionally, biomarker data were missing for one participant in the intervention group due to absence at T1, although his questionnaire data was included as he completed it remotely. Another participant, who had not yet completed hisT1 questionnaire before viewing the toolbox film, was excluded from the questionnaire analysis as this might have influenced his answers. Demographic characteristics are presented in Table 2.

**Table 2:** Demographic characteristics at baseline.

Characteristics		Intervention group	Control group
		(*n* = 25)	(*n* = 23)
Age (years), median (IQR)		47.0 (27.5–55.0)	41.0 (31.0–55.0)
Fitzpatrick Skin phototypes, *n* (%)	I		
	II	17 (68.0%)	13 (56.5%)
	III	8 (32.0%)	7 (30.4%)
	IV		2 (8.7%)
	V		1 (4.3%)
Sector, *n* (%)	Arboricultural work	4 (16.0%)	4 (17.4%)
	Ground-and demolition work	10 (40.0%)	12 (52.2%)
	Carpentry	11 (44.0%)	7 (30.4%)
Employment in sector (years), median (IQR)		25.0 (6.3–34.0)	15.0 (7.0–27.0)
Days between T0 and T1, mean (Min-Max)		42.5 (42–43)	41.8 (41–45)

IQR: interquartile range, T0: baseline, T1: 6 weeks after baseline.

### 3.1 Changes in relative cUCA as a biomarker of internal UVR exposure

The results of the LMM analysis showed significant differences in relative cUCA between the intervention and control group for the cheek location (*p* < 0.001). For the neck location the differences were less pronounced and not statistically significant (*p* = 0.085) (Table 3).

**Table 3:** Effects of intervention on the main outcome (relative cUCA) for both skin locations.

Comparison groups	Effect (B)	95% CI	*p*-value
Intervention vs. control (cheek)	−0.065***	−0.101– −0.029	<0.001
Intervention vs. control (neck)	−0.032	−0.068–0.004	0.085

CI = confidence interval, relative cUCA = relative cis urocanic acid (cUCA/total UCA). ***p < 0.001. *p*-values of <0.05 are considered statistically significant.

**Figure 3:**
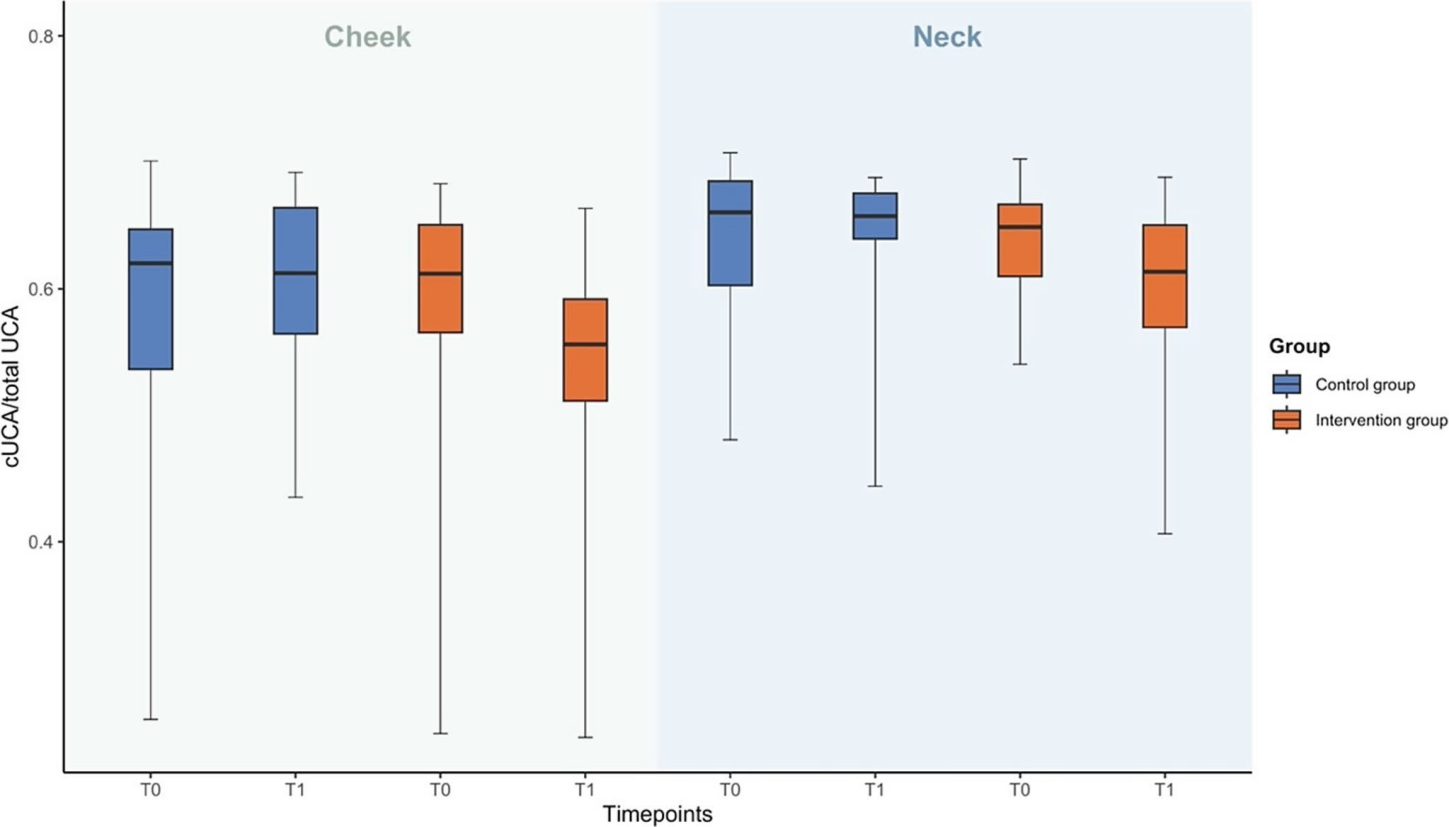
Descriptive data on relative cUCA in the intervention and control group and on different sample locations (cheek and neck) at T0 and T1.

A difference in relative cUCA was found between the sample locations (Table 3), with higher concentrations observed in the neck compared to the cheek in both groups (Figure 3).

### 3.2 Questionnaires

#### 3.2.1 Sun-protective behavior

A significant difference was observed between intervention and control group when a mixed model analysis was applied (Table 1). Sun-protective behavior improved in the intervention group, whereas the behavior in the control group remained unchanged.

Detailed descriptive information can be found in Supplementary Table S1.

#### 3.2.2 Workplace encouragement and facilitation of sun protection

No significant differences were observed between intervention and control group when a mixed model analysis was applied (Table 1). Descriptive data on workplace encouragement for sunscreen use increased in both groups, whereas encouragement for sun-protective clothing and shade-seeking remained unchanged. Detailed descriptive information can be found in Supplementary Table S1.

#### 3.2.3 Knowledge of sun-protective behavior and risk factors

No significant differences were observed between intervention and control group when a mixed model analysis was applied (Table 1). Descriptive data on sun-protective behavior showed a decrease in the intervention group in recognizing sun exposure as a primary cause of skin cancer, while the awareness in the control group slightly improved. The control group showed a greater increase in awareness regarding when to consult a doctor for suspicious skin lesions compared to the intervention group. Both groups showed improved understanding regarding sunscreen use, but the intervention group experienced a slight decline in recognizing the need for sun protection on cloudy days. Detailed descriptive information can be found in Supplementary Table S1.

#### 3.2.4 Attitude/motivation towards sun protection

No significant differences were observed between intervention and control group when a mixed model analysis was applied (Table 1). Descriptive data showed higher sunscreen use at baseline in the control group compared to the intervention group. Over time, an increase in sunscreen use was observed in both groups. Remaining unsure about using sunscreen decreased in the intervention group but remained stable in the control group. In the intervention group, the use of sun-protective clothing slightly decreased, while it slightly increased in the control group. In both groups, a decline in those who had never considered using sun-protective clothing was observed, with the intervention group showing an increase in those deciding to use it. Uncertainty about sun-protective clothing stayed stable in the intervention group but increased in the control group. Attitudes towards sunscreen remained consistent, with most participants rejecting the idea that sunscreen is not regarded as masculine. Motivation from employer-provided sunscreen increased in both groups, and overall motivation for sun protection remained high. Detailed descriptive information can be found in Supplementary Table S1.

#### 3.2.5 Post intervention evaluation

Based on descriptive data, a higher proportion of participants in the intervention group reported no sunburn compared to the control group, with both groups experiencing similar rates of frequent sunburn. In the intervention group, 24% experienced one or more sunburns. In the control group, 37% experienced one or more sunburns during the study period. Concerning toolbox satisfaction, most participants in the intervention group considered the availability of sunscreen the most beneficial feature, while fewer found the film and sunscreen equally useful, and some did not find either feature helpful.

#### 3.2.6 Process evaluation

Regarding recruitment and reach, of the 15 companies approached, five agreed to participate, resulting in a target audience of 180 outdoor employees, with 60% expressing interest in participation. Loss to follow-up was 11% in the intervention group (*n* = 3) and 12% in the control group (*n* = 3), leaving 25 participants in the intervention group and 23 in the control group. Post-study interviews revealed high satisfaction with sunscreen distribution and interactive discussions, and moderate to low satisfaction with the toolbox film. Detailed description can be found in Supplementary material S2.

## 4 Discussion

The toolbox intervention among male construction and arboricultural workers effectively reduced internal UVR exposure. These findings align with the improved sunscreen use observed in the intervention group. The intervention, however, did neither lead to improvements in the use of sun-protective clothing or shade-seeking behavior nor workplace encouragement, knowledge and attitudes about risk factors and sun-protective behavior.

This study confirms relative cUCA as a valuable marker to assess internal UVR exposure (28, 30). Unlike its trans-isomer UCA, cUCA is not endogenously present in the skin but is formed exclusively upon exposure to UVB radiation. Photoisomerization of trans-isomer UCA is a physical reaction that occurs independently of individual characteristics, making cUCA a highly specific marker of UVR exposure (31–36). As reported in our previous research in outdoor workers, we found that differences in relative cUCA were effective in distinguishing between more and less UVR exposed skin areas (i.e., forehead versus the retro-auricular region) (28). Notably, at baseline and follow up, the relative cUCA levels were higher in the neck compared to the cheek in both groups. This may reflect work-related postures and the orientation of body sites with respect to the sun, which influence internal UVR exposure. Moreover, unlike the cheek, the change in cUCA in the neck in the intervention group was lower. This may suggest that participants habitually overlooked applying sunscreen to the neck, despite receiving instructions. This is in line with findings by Azurdia et al., who reported that the posterior and lateral neck are often missed or inadequately covered with sunscreen (37).

Importantly, the intervention had a significant effect on relative cUCA levels at the cheek but not at the neck. These findings emphasize the importance of not only sunscreen application but also ensuring it is applied over all sun exposed areas (26–28). In our study, participants particularly appreciated the ease and pleasantness of using the spray sunscreen. This is encouraging, as outdoor workers often avoid sunscreen due to unmet needs for easy application and fast absorption (12). However, managers expressed concerns about the spray sunscreen costs and are considering more affordable alternatives for potential future use. One participant refused to use sunscreen, and some non-participating workers perceived it as harmful, contributing to their non-participation. This reflects misconceptions about sun protection among outdoor workers, similar to those identified in a previous study (38). Fewer sunburns were reported in the intervention group, but overall occurrences in both groups were low, likely due to the cool, rainy weather in North-Holland during the first half of the study period (39).

In contrast to the improvements in sunscreen use observed after the intervention, further efforts are needed to reduce sun exposure through seeking shade or wearing protective clothing, primarily by optimizing the workplace environment and practices. Interviews with participants and their employers revealed barriers such as impractical shade structures and discomfort with covering the skin. This finding aligns with a study by Jakobsen et al., which found that increased sun protection among Danish outdoor workers was mainly due to sunscreen use rather than other measures (40). In contrast, another study among Canadian outdoor workers, reported high sun-protective clothing and shade use, possibly due to more effective awareness campaigns (41). Similarly, a controlled intervention study by Babazadeh et al., (42) which included multiple educational sessions and incorporated skin cancer statistics, showed improvement in all aspects of sun-protective behavior in Iranian farmers. The observed differences in intervention effectiveness between studies suggest that multicomponent interventions and a better understanding of the risk perception of outdoor workers are crucial when developing interventions aimed at behavioral change. Risk perception refers to how individuals subjectively judge potential health risks they are exposed to and whether they will take action to protect their health, all of which are influenced by the social and cultural context (43, 44).

Outdoor workers perceived that this intervention did not improve the workplace efforts to facilitate and promote the use of sun-protective clothing and shade. Participants suggested introducing an internal coordinator to oversee adherence. Another potential solution could involve instituting a mandatory sun protection policy, though its effectiveness varies (13, 45, 46). Our findings highlight the challenges in improving sun protection habits and underscores the need for coordinated efforts involving employers, employees, healthcare professionals, and policymakers (10, 13, 17).

A potential explanation for the decrease in skin cancer risk awareness after the intervention is the film’s lack of more detailed risk information and its limited impact. Manager feedback indicated the film was perceived as monotonous and failed to convey the seriousness of sun exposure risks. After the intervention, self-reported uncertainty about the role of sun exposure in skin cancer and the need for protection on cloudy days increased. While participants often acknowledged the film’s content, they did not act on it. Suggested improvements included incorporating morbidity and mortality statistics, detailed guidance on recognizing symptoms, and depicting a person with a visible skin cancer lesion to enhance the film’s impact.

The strengths of this study include an objective biomarker for UVR internal exposure, prospective design and inclusion of both control and intervention groups. The participants represented a broad age range, with relatively balanced job tasks between the intervention and control groups. Sampling of relative cUCA was feasible, comparable to the findings in our previous pilot study (15). Lastly, this toolbox was multicomponent, not only providing sunscreens but also offering an educational film and an interactive discussion.

Various limitations should however be considered. Firstly, probability sampling and randomization were unfeasible due to recruitment challenges; however, control and intervention groups were matched by worksites and job tasks to mitigate bias. Secondly, grouping all participants from the same company to prevent contamination may have introduced bias. Thirdly, for secondary outcomes, self-reported behavioral outcomes may be subject to recall errors and social desirability. Fourthly, the power calculation was based on a proxy for our primary outcome (self-reported UVR exposure), as we did not find a study with an identical objective marker for UVR exposure. Finally, we included only Dutch speaking male participants, predominantly with skin phototypes II and III, which limits generalizability.

In the control group, three participants had Fitzpatrick skin types IV and V. The inclusion of these individuals may have influenced the results, as previous studies have shown that fair-skinned individuals exhibit a higher conversion of trans-to cis-UCA (47). A post-hoc sensitivity analysis excluding these participants confirmed the positive effect of the intervention, even more it yielded also a significant effects at the neck location (−0.040 (95% CI: −0.076– −0.005)). This might be caused by other biological mechanisms and/or other sun protective behavior (47).

### 4.1 What is next?

Future research should evaluate the long-term effectiveness of the toolbox using randomized designs, incorporating feedback from participants and managers to improve the educational film, and employing comprehensive strategies to foster lasting behavioral changes and sun safety culture. Besides skin health, the health of the eyes and heat stress should also be considered for future prevention strategies (48). Additionally, developing and validating a Dutch-language questionnaire, as well as translating it into multiple languages, could enhance future intervention evaluations.

### 4.2 Conclusion

The sun-safety risk communication toolbox showed a positive effect in reducing internal UVR exposure, consistent with increased self-reported sunscreen use among outdoor workers in the intervention group. This study confirms cUCA as a valuable marker in sun-safety risk communication. However, the toolbox did not significantly affect workplace encouragement, knowledge and attitudes regarding occupational sun exposure risks. These mixed results highlight challenges in changing sun safety behaviors in a working context.

## Data Availability

The datasets presented in this article are not readily available because datasets will remain within the research institute. Requests to access the datasets should be directed to f.l.deboer@amsterdamumc.nl.
