# Supplementary material for: Evaluation of a toolbox for the prevention of skin cancer among outdoor workers: an intervention study
Source: Front Public Health. 2025 Jun 9;13:1579180. doi: 10.3389/fpubh.2025.1579180 (PMC12184205; doi:10.3389/fpubh.2025.1579180)
Supplement: Supplementary file 1 [file Table_1.docx]

|  |  | T0 |  | T1 |  |  |
| --- | --- | --- | --- | --- | --- | --- |
| Question | Answer | Intervention | Control | Intervention | Control | |
|  |  | (n = 25) | (n = 22) | (n = 25) | (n = 22) | |
|  |  | T0 |  | T1 |  | |
| Sunscreen applications per week in the last week | 0 | 21 (84%) | 17 (77%) | 3 (12%) | 16 (73%) | |
|  | 1 | 1 (4%) | 2 (9%) | 2 (8%) | 2 (9%) | |
|  | 2 | 3 (12%) | 1 (5%) | 7 (28%) | 1 (5%) | |
|  | ≥3 | - | 2 (9%) | 12 (52%) | 3 (13%) | |
| Sunscreen applications per day | 0 | 10 (40%) | 12 (55%) | 1 (4%) | 11 (50%) | |
|  | 1 | 7 (28%) | 9 (41%) | 4 (16%) | 10 (46%) | |
|  | 2 | 8 (32%) | 1 (5%) | 12 (48%) | - | |
|  | ≥3 | - | - | 8 (32%) | 1 (5%) | |
| Workplace encouragement for sunscreen application  *(n = 21 in control group for T1)* | Yes | 12 (48%) | 9 (41%) | 16 (64%) | 12 (55%) | |
|  | No | 13 (52%) | 13 (59%) | 9 (36%) | 9 (41%) | |
| Workplace encouragement for sun-protective clothing | Yes | 9 (36%) | 4 (18%) | 8 (32%) | 6 (27%) | |
|  | No | 16 (64%) | 18(82%) | 17 (68%) | 16 (73%) | |
| Workplace encouragement for working in the shade  *(n = 21 in control group for T1)* | Yes | 13 (52%) | 10 (46%) | 14 (56%) | 10 (46%) | |
|  | No | 12 (48%) | 11 (50%) | 11 (44%) | 12 (55%) | |
| ‘Applying sunscreen once a day is enough to prevent sun damage’ | Correct | 20 (80%) | 11 (50%) | 24 (96%) | 13 (59%) | |
|  | Incorrect | 1 (4%) | 3 (14%) |  | 5 (23%) | |
|  | I don’t know | 4 (16%) | 8 (36%) | 1 (4%) | 4 (18%) | |
| ‘If I have a suntanned skin, I no longer need to apply sunscreen’ | Correct | 23 (92%) | 14 (64%) | 23 (92%) | 18 (82%) | |
|  | Incorrect |  | 3 (14%) | 1 (4%) | 1 (5%) | |
|  | I don’t know | 2 (8%) | 5 (23%) | 1 (4%) | 3 (14%) | |
| ‘If it’s cloudy, I still need to apply sunscreen and/or wear sun-protective clothing’ | Correct | 14 (56%) | 7 (32%) | 13 (52%) | 12 (55%) | |
|  | Incorrect | 6 (24%) | 8 (36%) | 5 (20%) | 6 (27%) | |
|  | I don’t know | 5 (20%) | 7 (32%) | 7 (28%) | 4 (18%) | |
| ‘Sun exposure is the main cause of skin cancer’ | Correct | 20 (80%) | 9 (41%) | 17 (68%) | 10 (46%) | |
|  | Incorrect |  | 3 (14%) | 2 (8%) | 1 (5%) | |
|  | I don’t know | 5 (20%) | 10 (46%) | 6 (24%) | 11 (50%) | |
| ‘I know when to see a doctor if I have a suspicious spot on my skin’ | Yes | 11 (44%) | 7 (32%) | 16 (64%) | 13 (59%) | |
|  | No | 11 (44%) | 9 (41%) | 5 (20%) | 4 (18%) | |
|  | I don’t know | 3 (12%) | 6 (27%) | 4 (16%) | 5 (23%) | |
|  |  |  |  |  |  | |
| Sunscreen | Never thought about it | 2 (8%) | 4 (18%) |  | 2 (9%) | |
|  | Unsure if I will use it | 6 (24%) | 4 (18%) | 1 (4%) | 4 (18%) | |
|  | Decided not to use it |  | 2 (9%) |  | 1 (5%) | |
|  | Decided to use it | 8 (32%) | 2 (9%) | 15 (60%) | 6 (27%) | |
|  | Already use it | 9 (36%) | 10 (45%) | 9 (36%) | 9 (41%) | |
| Sun-Protective clothing  *(n = 21 in control group for T0)* | Never thought about it | 7 (28%) | 9 (41%) | 5 (20%) | 6 (27%) | |
|  | Unsure if I will use it | 9 (36%) | 4 (18%) | 9 (36%) | 6 (27%) | |
|  | Decided not to use it | 1 (4%) | 2 (9%) |  | 3 (14%) | |
|  | Decided to use it | 4 (16%) | 3 (14%) | 8 (32%) | 3 (14%) | |
|  | Already use it | 4 (16%) | 3 (14%) | 3 (12%) | 4 (18%) | |
| ‘It motivates me to use sunscreen when my employer provides it’ | Yes | 18 (72%) | 12 (55%) | 22 (88%) | 16 (73%) | |
|  | No | 7 (28%) | 10 (46%) | 3 (12%) | 6 (27%) | |
| ‘It motivates me to use sunscreen when my employer pays regular attention to it’ | Yes | 19 (76%) | 14 (64%) | 18 (72%) | 15 (68%) | |
|  | No | 6 (24%) | 8 (36%) | 7 (28%) | 7 (32%) | |
| ‘It motivates me to use sunscreen when my colleagues use sunscreen as well’ | Yes | 12 (48%) | 12 (55%) | 14 (56%) | 10 (46%) | |
|  | No | 13 (52%) | 10 (46%) | 11 (44%) | 12 (54%) | |
| ‘It motivates me to use sunscreen as protection against skin cancer’ | Yes | 23 (92%) | 16 (73%) | 23 (92%) | 18 (82%) | |
|  | No | 2 (8%) | 6 (27%) | 2 (8%) | 4 (18%) | |

**Table S2.** *Workplace Encouragement*, Facilitation of Sun Protection, Knowledge about Sun-Protective Behavior and Risk Factors, Attitude/Motivation Towards Sun Protection
