# Supplementary material for: Evaluation of a toolbox for the prevention of skin cancer among outdoor workers: an intervention study
Source: Front Public Health. 2025 Jun 9;13:1579180. doi: 10.3389/fpubh.2025.1579180 (PMC12184205; doi:10.3389/fpubh.2025.1579180)
Supplement: Supplementary file 2 [file Data_Sheet_1.docx]

**Abbreviation Total nr of questions Category**

B 8 Sun Protective Behaviour

K 5 Knowledge of Sun Protective Behaviour and Risk Factors

A 8 Attitude/Motivation Towards Sun Protection

C 3 Corporate Culture and Facilitation of Sun Protection

A total sum score is calculated for category B (Sun Protective Behaviour). Each question yields up to 2 points, with higher scores indicating better behaviour. The score calculation is conducted using the criteria outlined in Table 1.

#### Table 1. Score Calculation

| Question | Answers | Points |
| --- | --- | --- |
| B1 | ❑ 0  ❑ 1  ❑ 2  ❑ 3  ❑ 4  ❑ 5  ❑ 6  ❑ 7 | 0  2/7  4/7  6/7  1 1/7  1 3/7  1 5/7  2 |
| B2 | ❑ 0  ❑ 1  ❑ 2  ❑ 3  ❑ 4 or more | 0  1/2  1  1 1/2  2 |
| B3 | ❑ Face  ❑ Neck  ❑ Hands and arm | 0 options selected: 0  1 option selected: 2/3  2 options selected: 1 1/3  3 options selected: 2 |
| B4-B6 | ❑ Yes  ❑ No  ❑ Sometimes | 2  0  1 |
| B7, B8 | ❑ Yes  ❑ No | 2  0 |
