# Supplementary material for: Evaluation of a toolbox for the prevention of skin cancer among outdoor workers: an intervention study
Source: Front Public Health. 2025 Jun 9;13:1579180. doi: 10.3389/fpubh.2025.1579180 (PMC12184205; doi:10.3389/fpubh.2025.1579180)
Supplement: Supplementary file 3 [file Data_Sheet_2.docx]

## **Process Evaluation**

A process evaluation, according to the Linnan & Steckler model, was conducted to assess the design and implementation of the sun-protective toolbox among construction and green sector workers (1). This model assesses recruitment, reach, dose delivered, dose received, fidelity, intervention satisfaction and context.

#### **Recruitment and Reach**

Participants were recruited at organisational and individual levels with a consistently applied recruitment process across all levels. Participating companies were recruited by the researchers through direct contact with individual contractors via telephone calls or email correspondence. The companies were selected using a convenience sampling approach, partly based on their participation in previous research and partly due to their location within the same regional area. All potential participating companies received an email detailing the study's aim and methods and an informative flyer to share with each potential participant. However, we cannot guarantee the distribution of information within the companies. One company was informed about the study protocol through an informative meeting.

The reach of our intervention represents the number of participating companies compared to the number of companies approached. One of the researchers collected data on the number of companies approached, companies that reacted, companies that agreed to participate, and reasons for non-participation. Several subgroups were less likely to participate. Some companies declined to participate due to a lack of time, with non-participation being higher among smaller companies with limited staff. Companies already engaged in sufficient sun protective behaviour did not participate as they believed that there was little room for further improvement by participating in our intervention. Larger, well-known companies were also underrepresented in the study sample due to non-responsiveness to our inquiries. Additionally, one company consisted primarily of non-Dutch-speaking workers, thus meeting one of our exclusion criteria and limiting the reach of our intervention to non-Dutch-speaking outdoor workers.

Ultimately, out of the 15 companies approached, 5 agreed to participate. Each company employs approximately 25-70 people, totalling around 250 employees. Only outdoor working staff were considered for inclusion; approximately 10-30% of the employees in each company were office staff and hence were excluded. Additionally, specific subdivisions, such as sewer service divisions, were excluded due to their limited hours of outdoor work. In one case, we only contacted the nearest working location of a company, resulting in a small target audience of 6 interested participants. Altogether, this led to a target audience of approximately 180 employees.

After the initial inquiry with the 5 companies, we received a list of employees who expressed interest in participating. About 60% of the target audience was interested in participating, not including the company for which we only contacted one location. Reasons for non-participation were not explored in all cases due to the differences in recruitment methods, making it unfeasible to collect this information uniformly. In cases where reasons were investigated, the main arguments included a lack of time, lack of interest/urgency, or aversion to using sunscreen. Therefore, these subgroups, which were less inclined to participate, are underrepresented in our study population. The subset that is specifically unwilling to use sunscreen represents a missed opportunity for potential improvement of sun-protective behaviour following our intervention. Furthermore, participants' voluntary decision to join the study may unavoidably introduce self-selection bias in our study sample, potentially compromising its representativeness for the entire population.

Eligibility was revised according to the inclusion and exclusion criteria described elsewhere (see ‘Methods’), leading to roughly 30 exclusions, primarily due to insufficient hours of outdoor work per day. Additionally, one female employee was excluded, and another employee was excluded for being too young (15 years old). Female participants were excluded, as construction workers are predominantly male. However, this exclusion also limits the reach of our study, as it prevents the evaluation of the toolbox in this subgroup. Among the eligible target audience, 6 employees were absent at data collection due to traffic, illness, or not working that day.

#### **Dose Delivered and Received**

Our study protocol ensured that the toolbox intervention was effectively delivered to all participants. We utilised an attendance sheet with a checklist to monitor sunscreen distribution among participants. To guarantee total attendance, we reviewed the attendance sheet again before showing the toolbox film.

Loss to follow-up (n = 6) was 11% in the intervention group (n = 3) and 12% in the control group (n = 3), leaving 25 participants in the intervention group and 23 in the control group. After baseline data collection, 1 participant in the intervention group was excluded from both questionnaire and biomarker analyses due to reluctance to participate. The remaining 5 lost participants were absent at the second appointment due to illness or holidays. This results in a high dose received, considering the mild attrition. However, the dose received in a broader sense, meaning the extent to which participants are receptive to the intervention, depends on multiple factors. Firstly, receptiveness to education and subsequent improvement in sun safety behaviour depends on the individual's stage of behavioural change (2). Developing stage-appropriate preventive measures and identifying an individual's stage before applying a preventive measure could improve the dose received in future studies. Secondly, the moderate amount of sunshine during the first half of the study period may have reduced the incentive to adopt sun protective measures and, therefore, might have reduced the dose received. This, however, applies to both the intervention and the control group. Our study aimed to estimate the dose received through skin biomarker and questionnaire analyses, comparing the intervention and control groups.

#### **Fidelity**

Our research was conducted as planned, with our methodology aligning with the initial plan outlined before the intervention began. The only modification was due to challenges in recruiting sufficient participants. Initially, we focused solely on construction workers; however, when it became evident that not enough eligible or willing construction companies could be recruited, we broadened our scope to include companies engaged in green work. Despite differences in job roles between these sectors, both involve outdoor work for at least four hours daily, making them appropriate risk groups for our study's target audience.

#### **Satisfaction and Context**

Satisfaction with the intervention was evaluated through post-study interviews with four randomly selected participants—two from each company in the intervention group—and their respective managers. Overall, satisfaction with the sunscreen and interactive discussion was high, while satisfaction with the toolbox film ranged from moderate to low. Both participants and managers would recommend the toolbox to others, emphasising the importance of understanding the risks of inadequate sun protection and valuing the provided resources and information for adequate sun safety. They also advocated for company-wide implementation of the toolbox.

In interviews with participants, the toolbox was reported to effectively raise sun risk awareness and subsequently improve sunscreen application, primarily to prevent painful sunburn and minimise skin damage and skin cancer. The sunscreen spray was particularly well-received for its ease of use. Participants recommended making sunscreen available at work, noting the need for company-wide initiatives to encourage sun protection and highlighting personal responsibility for on-location refills. However, managers expressed concerns about the spray sunscreen costs and are considering more affordable alternatives for potential future use.

While increased sunscreen application was objectified through questionnaires, our results do not support an improvement in awareness of skin cancer risks. One potential explanation for this finding could be that the toolbox film did not provide detailed risk information and had an insufficient impact. Managers indicated that the film was monotonous and did not adequately emphasise the gravity of sun exposure risks. It was also noted that risk awareness was low and that acquired knowledge quickly faded; acknowledgement of the information presented in the film was noted, yet participants admitted that they often failed to act on it. Suggested improvements for the film included integrating morbidity and mortality statistics, a detailed depiction of the severity of skin cancer diagnoses and treatments, and comprehensive information on recognising relevant symptoms.

Additional motivators for participants included participation in a study and educational input from a researcher with a medical background. Managers agreed, indicating that prior interventions aimed at enhancing sun risk awareness were less effective when implemented by the company itself, primarily due to the absence of a medical perspective and underlying rationale.

Participants also mentioned barriers to deploying other sun protection methods, such as seeking shade or wearing protective clothing and helmets. Firstly, participants mentioned the absence and impracticality of shade structures. Employees engaged in groundwork reported that the usage of shade structures is unworkable due to large working fields and the need to relocate. Rescheduling work to early mornings to avoid peak sun hours has been implemented before; however, this resulted in noise disturbance in residential areas and the risk of dangerous work situations due to workers’ fatigue. Secondly, the discomfort associated with covering exposed skin and scalp was discussed. Full-length trousers, long-sleeved shirts, and headwear were noted to be excessively warm and perspiring. It was also mentioned that increased corporate encouragement to acquire breathable protective clothing would enhance workers’ motivation to use it. Managers reported a sense of responsibility for facilitating sun protection but faced challenges implementing and enforcing these practices. Even when specific measures are facilitated, such as distributing wide-brimmed hats, they are not always adopted. Managers recommended employees articulate their needs and proactively approach their employer to request protective equipment, as they better understand their requirements than office staff. Managers also stated that employees should hold each other accountable for adhering to safety practices. Participants suggested that an internal coordinator could help maintain long-term adherence to sun protection measures. Another solution could be enforcing a mandatory sun protection policy. However, different studies report varying results on the effect of compulsory policies on improved sun protection (3–5). Our research highlights challenges in improving sun protection habits among individuals and businesses. Addressing these requires a comprehensive approach that involves employers, employees, healthcare professionals, and policymakers (5–7).

## **References**

1. Linnan L., Stecklar A. Process evaluation for public health interventions and research. In 2002.

2. Raihan N, Cogburn M. Stages of Change Theory [Internet]. 2023 [cited 2024 Jun 21]. Available from: https://www.ncbi.nlm.nih.gov/books/NBK556005/

3. Janda M, Stoneham M, Youl P, Crane P, Sendall MC, Tenkate T, et al. What encourages sun protection among outdoor workers from four industries? J Occup Health. 2014;56(1):62–72.

4. Woolley T, Lowe J, Raasch B, Glasby M, Buettner PG. Workplace sun protection policies and employees’ sun-related skin damage. Am J Health Behav [Internet]. 2008 [cited 2024 Jul 26];32(2):201–8. Available from: https://pubmed.ncbi.nlm.nih.gov/18052860/

5. Glanz K, Buller DB, Saraiya M. Reducing ultraviolet radiation exposure among outdoor workers: state of the evidence and recommendations. Environ Health [Internet]. 2007 [cited 2024 Jul 26];6. Available from: https://pubmed.ncbi.nlm.nih.gov/17686155/

6. Reinau D, Weiss M, Meier CR, Diepgen TL, Surber C. Outdoor workers’ sun‐related knowledge, attitudes and protective behaviours: a systematic review of cross‐sectional and interventional studies. British Journal of Dermatology [Internet]. 2013 May 1 [cited 2024 Jul 31];168(5):928–40. Available from: https://dx-doi-org.vu-nl.idm.oclc.org/10.1111/bjd.12160

7. Symanzik C, Ludewig M, Rocholl M, John SM. Photoprotection in occupational dermatology. 2023 [cited 2024 Jul 26];22:1213–22. Available from: https://doi.org/10.1007/s43630-023-00385-6
